# Economic evaluation of stormwater capture and its multiple benefits in California

**DOI:** 10.1371/journal.pone.0230549

**Published:** 2020-03-24

**Authors:** Sarah E. Diringer, Morgan Shimabuku, Heather Cooley

**Affiliations:** Pacific Institute, Oakland, California, United States of America; Universita degli Studi di Pisa, ITALY

## Abstract

Urban stormwater is increasingly being considered a viable alternative water supply in California and throughout the southwestern U.S. However, current economic analyses of stormwater capture do not adequately examine differences in stormwater project types and do not evaluate co-benefits provided by the projects. As a result, urban stormwater capture is undervalued as a water supply option. To advance economic analyses of stormwater capture, we determined the levelized cost of water in U.S. dollar per acre-foot of water supply (AF; 1 AF = 1233.5 m^3^) for 50 proposed stormwater capture projects in California, characterizing the projects by water source, process, and water supply yield. In addition, we incorporated reported co-benefits of projects into the analysis to determine the net benefit of proposed projects. Proposed urban stormwater capture projects were more expensive than non-urban stormwater capture projects on a per-volume basis ($1,180 per AF and $531 per AF, respectively); however, this was primarily driven by the relatively large size of the non-urban stormwater capture projects examined. When incorporating the limited number of reported co-benefits of the projects, the expected levelized cost of water from urban stormwater capture projects decreased dramatically. For projects that reported even a limited number of additional benefits, the net levelized cost decreased from $1,030 per AF to $150 per AF, with some of the projects demonstrating a net benefit. Thus, scaling urban stormwater capture projects to capitalize on economies of scale and incorporating co-benefits of projects can dramatically improve the economic feasibility of these projects. This work demonstrates that stormwater capture can present a cost-effective water supply option in California, and that beyond California, fairer comparisons among projects and inclusion of co-benefits can provide decision makers with adequate information to maximize investments in water management.

## Introduction

In urban areas, precipitation and excess irrigation that falls on impermeable or semi-permeable surfaces, such as roofs or roads, can run off into nearby channels and waterways, carrying oils, heavy metals, soil, salts, and other pollutants with it. This water, commonly referred to as urban stormwater, has historically been managed to mitigate flooding and surface water pollution by developing street curb water collection and sewer systems that quickly route the water away from urban centers and address the pollutants generated by stormwater runoff [1]. Most recently, an additional shift is occurring within stormwater management: with growing pressure on water supplies, communities in California and the southwestern U.S. are expanding stormwater management to include investment in stormwater capture as a means of augmenting water supplies. These capture projects range from small-scale rainwater harvesting to large aquifer recharge projects, and can simultaneously mitigate flood risk, reduce water quality impairments, and provide additional water supplies [2].

Studies demonstrate that there are substantial opportunities for capturing stormwater in California. A 2014 study estimated that capturing stormwater from paved surfaces and rooftops in urbanized Southern California and the San Francisco Bay Area could increase average annual water supplies by between 420,000 to 630,000 acre-feet (AF; 1 AF = 1233.5 m^3^) each year, representing between 6–10% of 2014 total water usage in those regions [3,4]. In addition, stormwater capture is becoming a central tenant in low-impact and sustainable development, through distributed rainwater and stormwater capture systems. For example, in 2011, the City of Los Angeles passed a low-impact development (LID) ordinance requiring stormwater runoff to be captured on-site to increase groundwater recharge and offset imported water needs [5].

To help realize this potential, the state is promoting stormwater capture efforts, with the State Water Board setting a goal to increase stormwater capture and use 500,000 acre-feet per year (AFY) over 2007 levels by 2020 [6] and by encouraging stormwater projects that include water supply benefits [5]. In addition, a 2018 draft report by the California Department of Water Resources (DWR) showed that the completion of planned, funded stormwater capture projects in the state would contribute 250,000 AFY of water supply from urban stormwater runoff by 2035, primarily through groundwater recharge [7]. As climate change continues to impact water supply reliability, there is need to accelerate investment in stormwater capture as an additional water supply and to overcome perceived and actual barriers to stormwater capture projects.

As entities explore stormwater capture, there is little information about the actual cost of building and maintaining stormwater capture systems over the lifetime of the projects. Previous work found that large stormwater capture projects (> 6,500 AFY) in California had a median levelized cost of $590 per acre-foot and smaller projects (≤ 1,500 AFY) had a median cost of $1,500 per acre-foot [8]. Similarly, Perone and Rohde (2016) determined the median cost of stormwater capture through managed aquifer recharge (MAR) projects at $1,550 per acre-foot and ranged dramatically, from $410 per acre-foot to $2,660 per acre-foot [9]. While this previous research demonstrates that stormwater capture is an economically viable water supply options, these studies were relatively limited in scope of stormwater capture projects considered and neither incorporated co-benefits of stormwater capture projects, such as reducing pollution in nearby waterways, avoiding the cost of Clean Water Act compliance, and minimizing flooding.

The research presented here builds on previous work by evaluating a larger number of stormwater capture projects with a more in-depth analysis of specific strategies employed and by incorporating several co-benefits into the economic analyses. In addition, stormwater capture projects include a wide range of processes, including centralized capture (such as stormwater conveyance and spreading basins) and decentralized capture that infiltrates runoff on site or uses it directly for irrigation or other non-potable sources. The term stormwater can be used to describe water that runs off impervious surfaces in urban landscapes and/or as runoff from non-urban areas, which is often also referred to as surface- or floodwater. Thus, “stormwater capture” can refer to a wide range of processes that vary in terms of costs and net benefits. Characterizing both urban and non-urban stormwater capture projects allows for greater understanding of the economics of individual strategies.

This research also seeks to advance economic analyses of stormwater capture by integrating project co-benefits to the extent practicable. In addition to improving water supply reliability, some stormwater capture projects can provide additional co-benefits, such as improving habitat, reducing urban temperatures and energy use, creating community recreation spaces, and increasing property values [10–13]. However, current methods for examining the costs of stormwater capture as a water supply are insufficient for effectively incorporating these multiple benefits. Analyses often attribute the entire cost of a project to a single benefit, which dramatically undervalues integrated projects and incentivizes single-purpose projects that optimize for water supply. By including the economic value of co-benefits provided by stormwater capture, projects can be more fairly compared, and the full benefits of these projects can more easily be realized by water agencies and the public they serve.

## Methods

### Data collection and characterization

The data collection method was informed by Perrone and Rhode (2016)’s “Benefits and Economic Costs of Managed Aquifer Recharge in California” [9]. Stormwater capture project data were collected from proposals submitted to California Propositions 84 in Rounds 1 and 2 in 2011 and 2013 respectively and 1E funding grants in Rounds 1 and 2 in 2013 and 2015 respectively through the California Department of Water Resources (DWR). Proposition 1E focuses funds on disaster preparedness and flood prevention. Proposition 84, in part, focuses on supporting planning and implementation for Integrated Regional Water Management (IRWM). The first two funding rounds of each of these propositions were selected for analysis because they contained financial information on capital costs, operation and maintenance (O&M) costs, and monetized benefits of proposed projects. Projects were included in the analysis if they: 1) contained a stormwater and/or floodwater capture component, 2) quantified the volume of water supply that would be obtained from the project, and 3) included expected capital and O&M costs. Both awarded and declined projects were considered for analysis.

There were 533 individual projects included in 75 submitted proposals. Of these projects, 435 projects did not include a stormwater and/or floodwater capture component (e.g., water efficiency or water reuse projects). An additional 46 projects did not quantify the expected volume of additional water supply and/or the monetary value of the water supply captured, and thus were not included in the analysis. This yielded a total of 52 projects for consideration in analyses. Two projects were removed as statistical outliers (see statistical methods below), yielding a total of 50 projects included in analyses. Applicable projects were examined in detail and data were collected manually, including applicant and funding information, location, project description, project design components, costs, and benefits report (Table 1; S1 Dataset). The benefits included in the analysis are those that were reported in the project proposals.

**Table 1:** Data collected from grant applications.

Data Categories	Specific Data Collected
**Applicant and Funding**	Funding Scheme
	Award Year
	Lead Agency
**Location**	DWR Planning Area
	DWR Hydrologic Unit
	Latitude
	Longitude
**Project Description**	Project Name
	Primary Project Goal (reported)
	Lifetime
**Project Design**	Site Status (Upgrade/Expansion, New Project)
	Process (Recharge, Storage, Conveyance, Treatment, Direct Use)
	Location Relative to Waterway (On-Stream, Off-Stream, Both)
	Urban vs. Non-Urban (Urban, Non-Urban, Both)
	Additional Water Source (if applicable: Floodwater, Surface Water, Wastewater, Combination)
**Costs**	Capital costs
	Operation and maintenance (O&M) costs
	Additional costs
**Benefits Reported**	Water Supply Yield (AFY, $)
	Stormwater Water Supply (AFY, $)
	Other Water Supply (AFY, $)
	Flood Damage Reduction ($)
	Water Quality ($)
	Energy or Electrical Savings ($)
	Community Recreation, Public Use, or Property Values ($)
	Habitat Value ($)
	CO_2_ Equivalents ($)
	Avoided Costs ($)

Individual projects were characterized based on design categories to better describe project types, including: (1) site status (i.e., new projects or expansions/upgrades to existing projects); (2) process (i.e., conveyance, storage, recharge, treatment, and/or direct use), (2) water source (urban or non-urban stormwater capture); (3) location relative to waterway (i.e., on stream, off-stream, or both); and (4) additional water source (i.e., surface water, flood water, and/or wastewater). Site status and process were determined through project descriptions in individual proposals. Categorization as urban stormwater projects compared to non-urban stormwater projects was determined through project descriptions or location based on GPS coordinates and the expected origin of the water to be captured. Projects were considered non-urban stormwater capture if urban runoff was incidental, rather than the predominant source of water, and the proportion of other water sources were not specifically reported as part of the broader project. Additional water sources were included separately from stormwater if the project proposal quantified the multiple sources of water separately.

**Fig 1:**
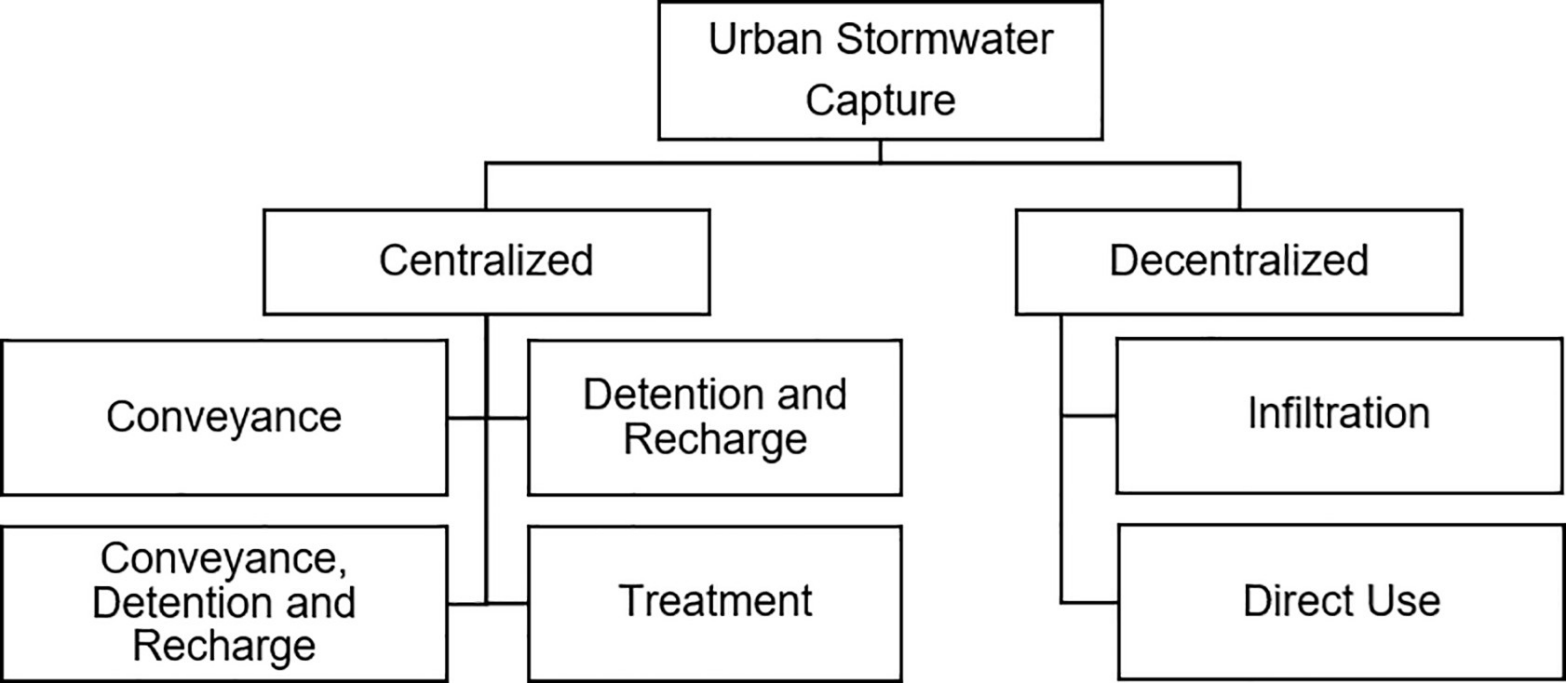
Stormwater typology for selected projects with the total number of projects included in the analysis for each project type.

State-wide efforts on stormwater capture are particularly focused on understanding urban stormwater capture and LID. Thus, the urban stormwater capture projects were further characterized for analysis as centralized or decentralized and by capture process (Fig 1). Decentralized stormwater capture refers to projects that capture dry or wet weather runoff on or adjacent to the parcel from which it originates, and centralized capture projects require natural or man-made conveyance for capture prior to storage or use. Centralized stormwater projects require several processes to capture, convey, and either recharge or use collected water. However, many projects only include specific components of the process. To understand the differences in cost among projects, they were separated by process for the analysis into conveyance; conveyance, detention, and recharge; detention and recharge alone; and treatment for use. Similarly, decentralized projects, or those that either infiltrate or collect water for use on site, can be categorized by processes or components, including for direct use (e.g., rain barrels or cisterns) or for infiltration (e.g., bioswales, permeable pavers, etc.). The selected decentralized projects all included a mixture of processes, and thus could not be further separated for this analysis.

### Economic analyses

The economic analyses were based on methods developed in the field of energy economics and modified to estimate the levelized cost of water in California [14]. This approach accounts for the full capital and operating costs of a project over its useful life and allows for comparisons among projects with different scales of operations, investment, and/or operating periods. It is particularly useful for comparing projects that achieve the same outcome where the outcome is challenging to monetize. In addition, this method is commonly used by water managers in California and throughout the country to compare alternative water supply options. The levelized cost of water (LC) in dollars per acre foot of water ($/AF) was determined as the annualized cost of each project ($/year) standardized by the reported annual water supply yield (WS) in acre-feet per year (AFY) (one acre-foot is equal to 1,233 m^3^). The annualized cost was calculated based on expected capital cost (C), annual operation and maintenance (O&M) costs, and a capital recovery factor (CRF), which is equivalent to an amortization factor in financial analyses (Eqs 1 and 2):

$$LC=\frac{[\sum_{t=1}^{n}(C_{t}+{O\&M}_{t})]*CRF}{WS}\;\mathrm{and}$$ (1)

$$CRF=\frac{{r(1+r)}^{n}}{[{r(1+r)}^{n}]-1},$$ (2)

where n is the useful life (years), t is the time since construction began (years), and r is the discount rate. We adopted a 6% discount rate based on the DWR proposal recommended discount rate.

The net levelized cost (NLC) in $/AF incorporated co-benefits reported in the project proposals by subtracting the monetized value of the co-benefit(s) in year t (CB_t_) from the total cost and standardizing the net benefit to the water supply yield (Eq 3).

$$NLC=\frac{[\sum_{t=1}^{n}{(C}_{t}+{O\&M}_{t}-{CB}_{t})]*CRF}{WS}$$ (3)

Benefits were determined from the reported value of monetized co-benefits, such as reductions in flood risk and/or energy cost. Monetized water supply benefits were not included in the net levelized cost analysis. Environmental and social costs and benefits were included in the analysis if they were monetized; additional benefits that were quantified in non-monetary units were not included in analysis. All costs have been adjusted for inflation and are reported in year 2018 dollars.

### Sources of uncertainty

There are several sources of uncertainty in the analysis. First, the cost and availability of these project options may vary according to local conditions and economic analyses for specific regions or projects should be based on site and project specifications. Second, the data collected represent proposed project costs, which may vary from true projects costs as a result of design changes, construction delays, regulatory and price changes, or other factors. While Perrone and Rohde (2016) demonstrated some similarities between proposed and actual annualized costs per recharge volume, additional research is necessary to determine the true costs of projects [9]. Third, the recharge volume can vary from proposed volumes due to, for example, annual variation in rainfall or operation at less than full capacity. These variations in recharge volumes affect the cost per unit of water supply and should be examined in more detail. Finally, quantification and monetization of benefits were estimated based on available methods and data and may not represent the total benefits and societal costs of these projects. For these reasons, the costs and benefits presented here should be used as a general guide for communities and decision makers to consider additional water supply investments.

### Statistical analyses

The levelized cost of water supply ($/AF) was log-transformed for statistical tests to yield a normal distribution. When log-transformed, these data do not differ significantly from a normal distribution (Shapiro Wilks, a = 0.05). Outliers were defined as greater than 1.5 times the interquartile range (IQR) above the third quartile or below the first quartile. Two projects were removed from the analysis as outliers. All statistical tests were performed in R: A language and environment for statistical computing (Version 1.1.463).

## Results

### Project characterization

**Fig 2:**
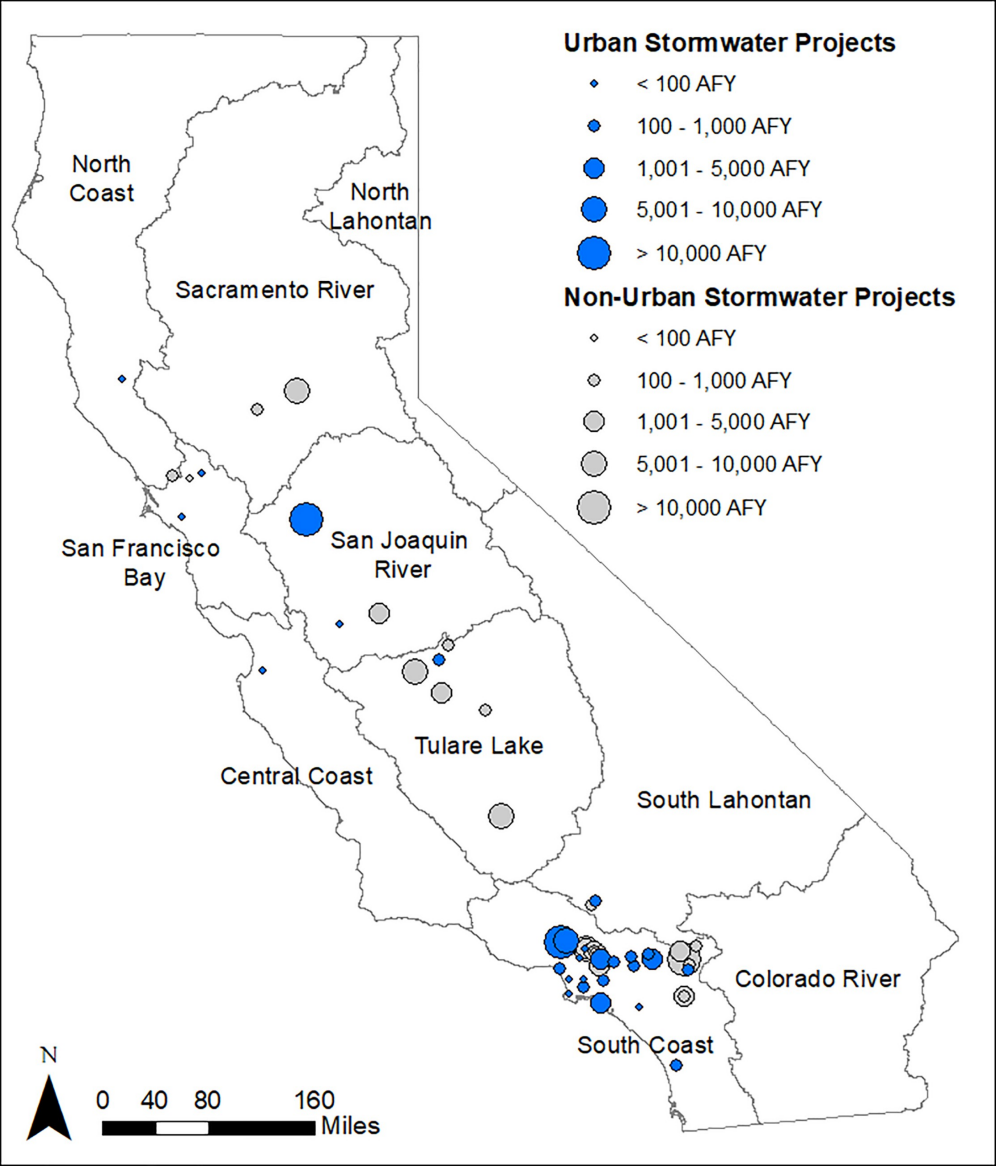
Project locations for 50 selected projects. Projects include 26 urban stormwater capture projects (blue) and 24 non-urban stormwater capture projects (grey). Project markers are proportional to expected total water supply yield in acre-feet per year (AFY). Maps were developed in ArcMap version 10.5. Base maps provided by California Natural Resources Agency Open Data Platform.

A total of 50 projects met the criteria for analysis, including 19 from proposition 1E and 31 from Proposition 84. Twenty-six of the proposed projects were designated as urban stormwater projects, with the remaining 24 projects as non-urban stormwater projects. The selected projects were located in eight different DWR hydrologic regions, but concentrated in the South Coast region of California, where 32 of the 50 stormwater capture projects and 19 or 27 urban stormwater capture projects were proposed (Fig 2).

Projects varied by 3 orders of magnitude in their total stormwater water supply yield (i.e., the water supply made available through stormwater capture per year over the lifetime of the project in AFY), ranging from 1.53 to 17,400 AFY, with a median of 552 AFY (Table 2). Urban stormwater capture projects reported smaller water supply yields, on average, than non-urban stormwater capture projects (375 AFY and 1,050 AFY, respectively), though urban stormwater capture projects also contained a greater range of reported water supply yields.

**Table 2:** Summary statistics describing stormwater water supply yield (AFY) and levelized cost ($/AF) for urban and non-urban stormwater projects.

	Min	25^th^ Percentile	Median	75^th^ Percentile	Max
**Stormwater Capture Water Volume (AFY)**					
Urban Stormwater (n = 26)	1.53	38.5	375	943	17,400
Non-Urban Stormwater (n = 24)	11.0	474	1,050	2,860	12,171
All Projects (n = 50)	1.53	163	552	1,650	17,400
** Levelized Cost of Water ($/AF)**					
Urban Stormwater (n = 26)	100	491	1,180	6,423	103,000
Non-Urban Stormwater (n = 24)	15.7	122	531	1,170	25,600
All Projects (n = 50)	15.7	246	816	2,560	103,000

### Costs of proposed stormwater capture projects

**Fig 3:**
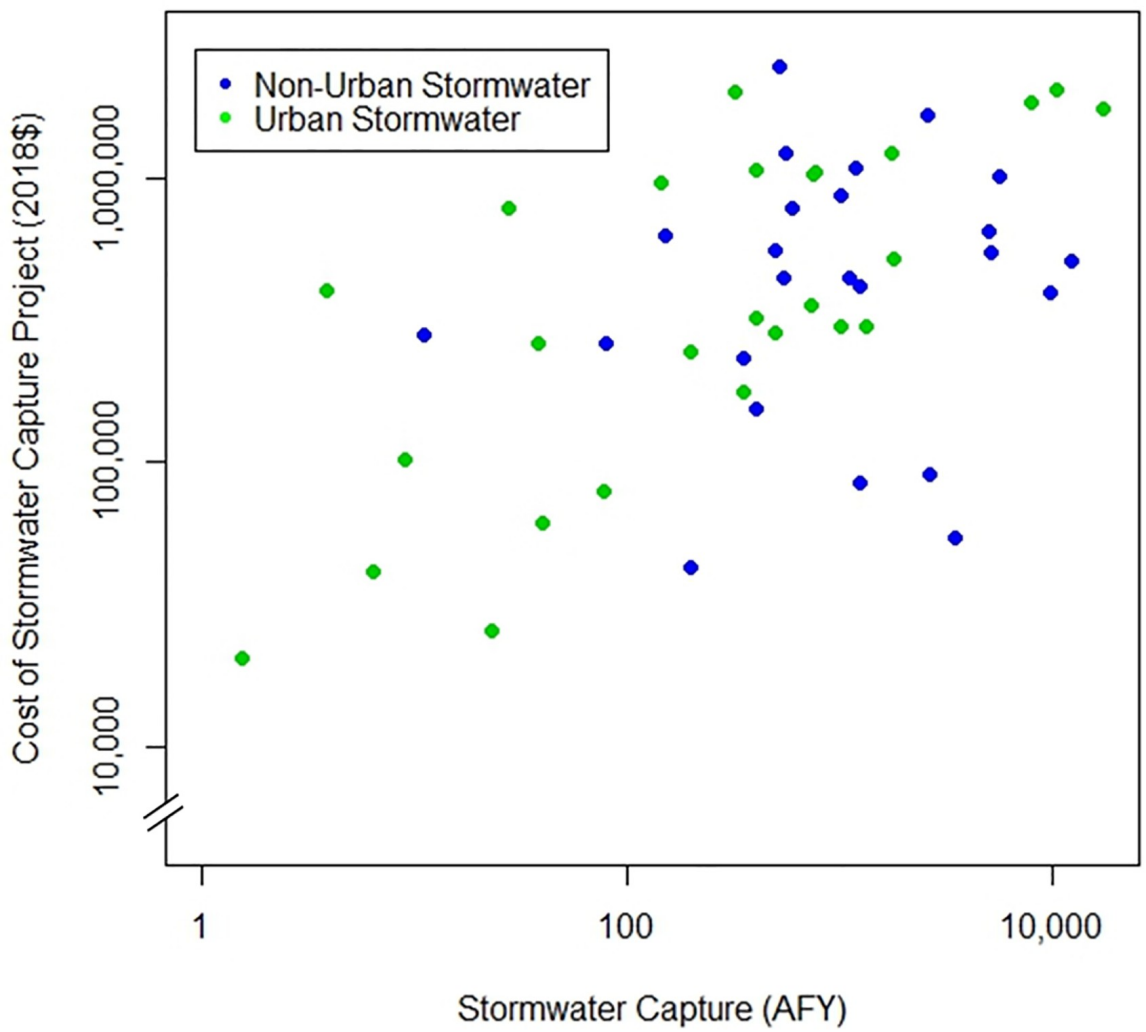
Scatter plot of stormwater capture volume (AFY) by levelized cost of water ($/AFY) in log-log scale for urban stormwater capture projects (green) and non-urban stormwater capture projects (blue).

The median levelized cost of water for the 50 projects examined was $816 per AF, ranging from $15.7 to $103,000 per AF. Projects exhibited strong economies of scale, with much lower levelized costs for larger projects compared to smaller projects (Fig 3). A multiple linear regression was calculated to predict the annualized project cost based on (1) the water supply yield, (2) stormwater source from urban or non-urban areas, and (3) site status (i.e., upgrade/expansion or new project). Model assumptions for normality and homoskedasticity were tested and verified visually. The results of the regression indicate that water supply yield and project location significantly influence the total project cost, with 35% of the variation in project cost accounted by water supply yield and location of the project (F(2,47) = 14.4, p < 0.001; Regression Results, S1 Dataset). Water supply yield alone explains 27% of the variation in the cost stormwater capture projects (F(1,48) = 18.7, p< 0.001), with the resulting model:

ln(annualizedprojectcost)=11.0+(0.29*ln(watersupplyyield)), (4)

demonstrating that a 10 percent increase in water supply yield results in a 2.8 percent increase in annualized project cost.

Inclusion of the water source and the site status did not have a significant impact on the levelized cost of water for these projects (Regression Results, S1 Dataset), and the model results were not improved by the inclusion of these variables (R^2^ = 0.34, F(4,45) = 7.19, p < 0.001). Thus, while the median cost of urban stormwater was 2.2 times greater per AF than that of non-urban stormwater projects ($1,180 and $531 per AF respectively), the increased cost was likely driven by project locations and water supply yields, rather than by the source of stormwater from either urban or non-urban areas.

### Urban stormwater capture projects

As urban stormwater is a specific focus of research, these projects were examined in more detail by project type (i.e., conveyance alone, conveyance and recharge, recharge alone, Decentralized projects, water treatment). A multiple linear regression was conducted to predict the annualized cost of urban stormwater projects based on the (1) water supply yield, (2) site status, and (3) stormwater project type. Water supply yield and stormwater project type were significant predictors of levelized cost of water for urban stormwater capture projects, and the model accounted for 68% of variance (F(6,19) = 11.5, p<0.001). For urban stormwater capture projects, annual water supply yield accounted for 51% of the variation in annualized cost of urban stormwater capture (F(1,24) = 28.0, p<0.001), with the resulting model:

ln(annualizedprojectcost)=10.5+(0.41*ln(watersupplyyield)) (5)

While this model can provide information on the impact of water supply yield on total project cost, it should be noted that this is not the minimum adequate model as stormwater project type was a significant predictor of project cost.

**Fig 4:**
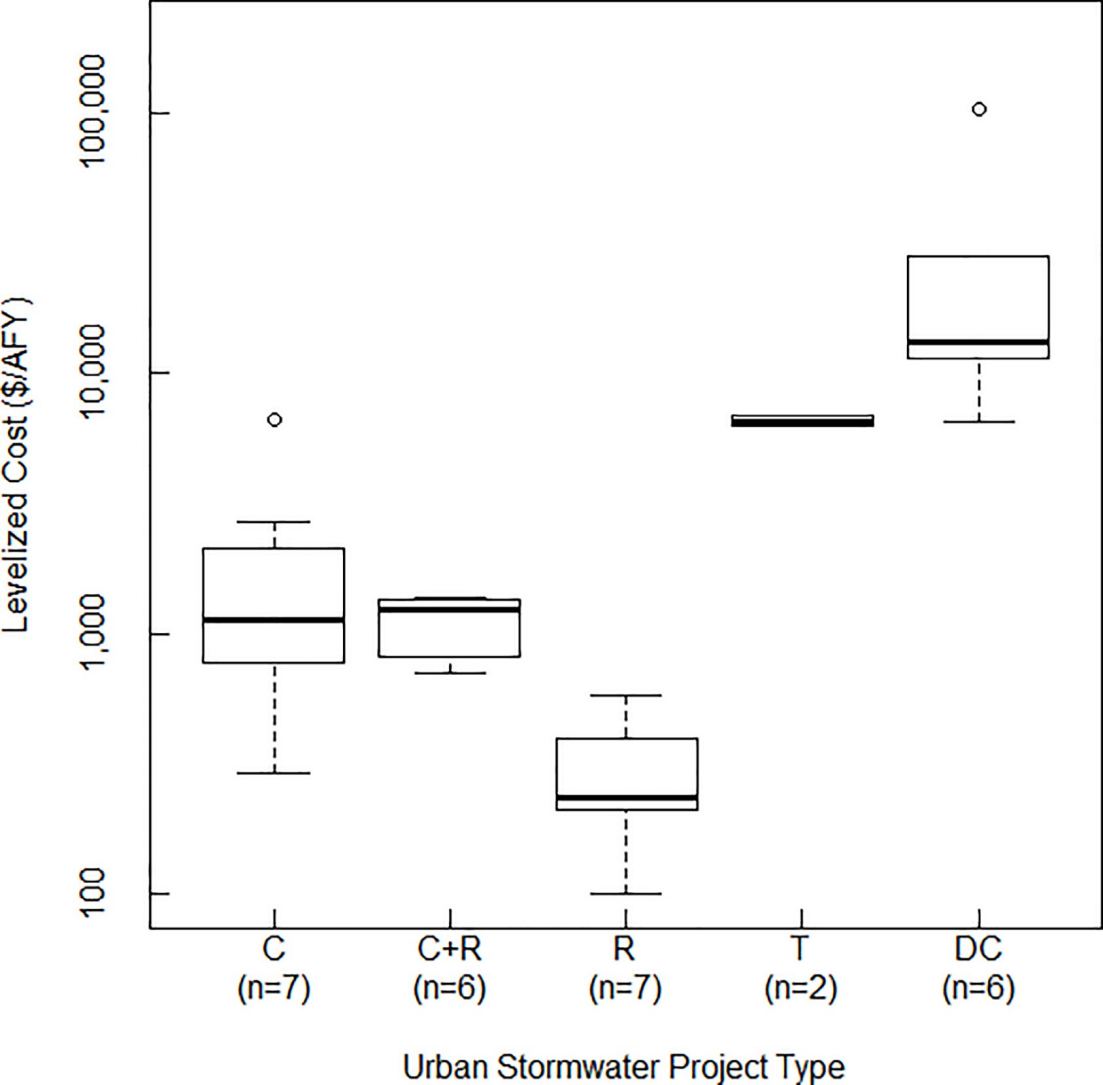
Boxplot of levelized cost of water ($/AFY) for five types of urban stormwater capture project types: Conveyance (C), conveyance and recharge (C+R), recharge (R), treatment (T), and decentralized (DC).

Decentralized (DC) stormwater capture projects had the highest median cost per acre foot of water ($13,300 per AF), while projects that included infrastructure for developing or expanding recharge (R) (e.g., retention or detention basins) had the lowest median levelized cost ($231 per AF) (Fig 4). In a comparison of conveyance (C), conveyance and recharge (C+R), and recharge projects (R), recharge projects were much less expensive than conveyance projects while conveyance and recharge projects were not substantially different than conveyance projects. This suggests that cost of conveyance is a substantial part of the overall cost of centralized stormwater capture projects.

### Multiple benefits of stormwater capture

Each project was required by the grant guidelines to demonstrate at least one quantifiable benefit. The IRWM program (funded through Proposition 84) required at least one quantifiable benefit from, for example, water supply reliability; stormwater capture, storage, clean-up, treatment, and management; or ecosystem and fisheries restoration and protection [15]. Proposition 1E allocated funds for stormwater flood management projects that were designed to “manage stormwater runoff to reduce flood damage and where feasible, provide other benefits” [16]. While it was not required that all proposals quantify or monetize the benefits of the projects, many of the project descriptions included additional co-benefits of the project.

**Fig 5:**
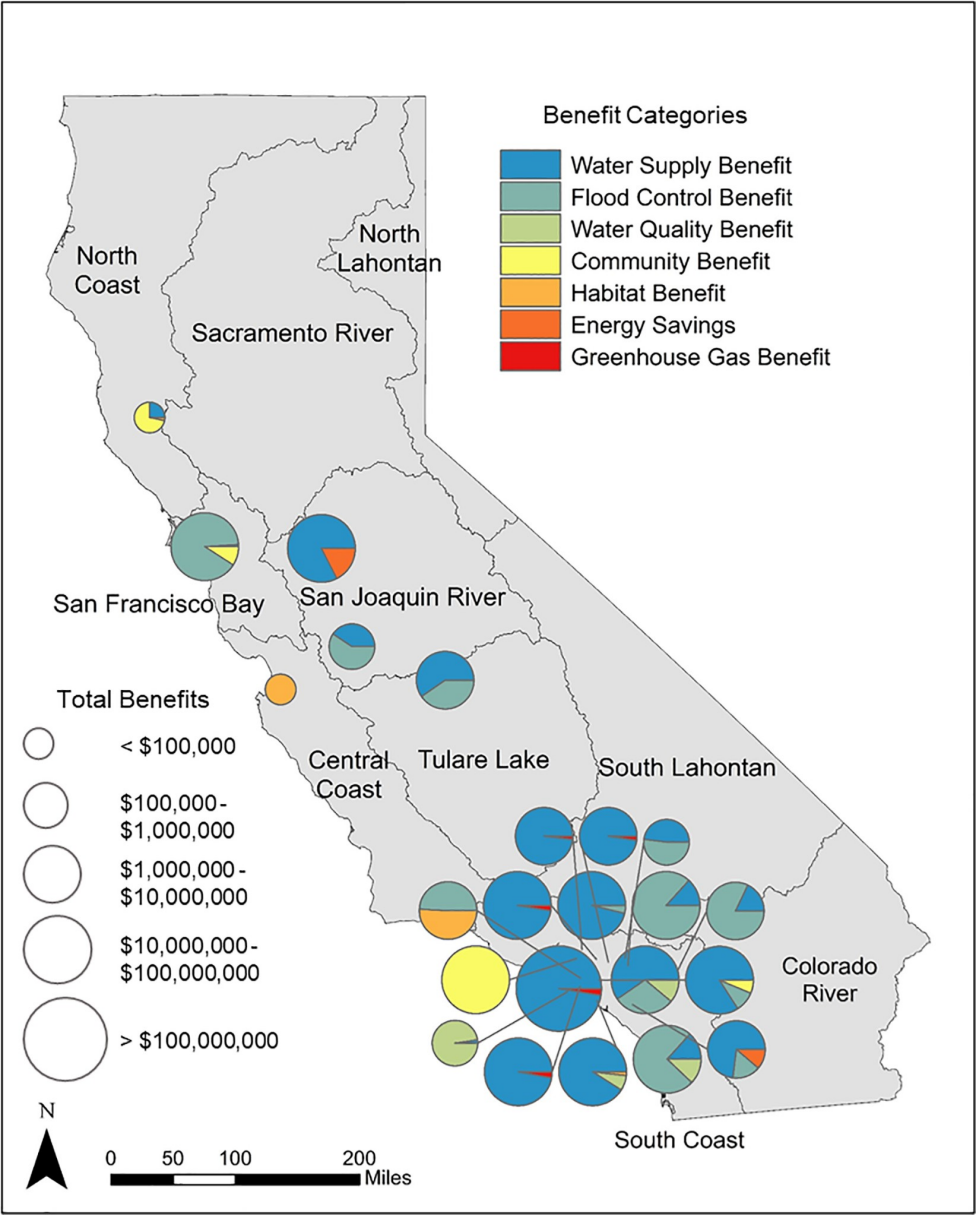
Value of reported multiple benefits for urban stormwater projects for projects with at least one co-benefit reported. Marker size represents the total benefits estimated for each project ($) and colors denote the benefit category. Maps were developed in ArcMap version 10.5. Base maps provided by California Natural Resources Agency Open Data Platform.

Projects were only included in the analysis if the volume of water supply yield in AFY was included, however, not all projects monetized the water supply yield. Twenty-three of the 26 urban stormwater capture projects monetized the volume of water in dollars, ranging from a total benefit of $365 to $12,800,000 per year (Table 3; Fig 5). Twenty-one of the 26 projects monetized at least one benefit beyond water supply, termed here as a co-benefit of the project for this analysis. The most commonly cited co-benefit was flood damage reduction (11 of 26 projects), following by greenhouse gas emissions reductions or sequestration (5 of 26 projects), habitat (4 of 26 projects), water quality (3 of 26 projects), and/or community benefits (including recreation and/or property values) (3 of 26 projects), and energy and/or electricity savings (2 of 26 projects). Additional project proposals included quantified co-benefits in physical terms (e.g., kilowatt hours of electrical savings or acres of greenspace created) but did not include these benefits in monetary terms, and thus could not be included in the analysis.

**Table 3:** Reported economic value of benefits in dollars per year for urban stormwater capture projects that reported at least one monetized benefit.

	Min	25^th^ Percentile	Median	75^th^ Percentile	Max
Water supply benefit (n = 23)	365	9,590	127,000	464,000	12,300,000
Flood damage reduction (n = 11)	487	10,204	45,800	216,000	3,830,000
Greenhouse gas emissions reduction or sequestration (n = 5)	6,250	14,000	31,500	57,000	316,000
Habitat benefit (n = 4)	46		1,290		9,230
Water quality benefits (n = 3)	8,180		52,900		132,000
Community benefits (n = 3)	17		39,400		194,000
Energy and/or electricity requirement reduction (n = 2)	9,870				67,500

The approach for monetizing benefits varied among agencies and projects (Table 4). For example, water supply benefits were monetized as revenue from sales of water to end users or avoided cost of purchasing imported supplies. Flood damage reductions were monetized in several ways, including as avoided flood damage to property, avoided emergency response costs, and reductions in insurance premiums.

**Table 4:** Benefit categories and selected metrics reported in proposals.

Benefit	Benefit Metric (2018 USD)
Water Supply	• Avoided cost of purchasing imported supplies • Cost savings for water users relative to the status quo • Revenue from sales of water to other users • Avoided operations and maintenance costs
Flood Damage Reduction	• Avoided flood damage to residential and non-residential properties • Avoided loss of revenue and wages from flood disruptions to business • Avoided emergency response costs • Reduced insurance premiums • Avoided public safety and health impacts
Water Quality	• Avoided cost of water treatment
Energy and/or Electrical Savings	• Avoided or reduced energy use from groundwater pumping or surface water transfers
Community Benefits	• Added public active and passive recreation space (acres of space) • Increased property values
Habitat	• Economic value of ecosystem services of wildlife habitat • Value of in stream flows
Greenhouse Gases Avoided	• Avoided greenhouse gas emissions (metric tons of CO2e per year) • Carbon sequestration (metric tons of CO2e per year)
Avoided Costs	• Avoided lowest-cost project alternative • Avoided operations and maintenance, including groundwater pumping

The primary goal of each project was determined from the proposals to examine the value of the benefit associated with the stated primary goal compared with the value provided by additional benefits. In 17 of 26 projects, the stated primary goal of the project (water supply, flood control, water quality, or other) provided the greatest economic benefit. In the remaining projects, other benefits provided greater economic value than the stated primary goal of the project (for example, a water supply project that derived the greatest economic benefit from reducing the risk of flooding). While Proposition 1E was primarily designed to support flood control projects, there was no apparent relationship between the proposition funding source and the monetized flood control benefit.

**Fig 6:**
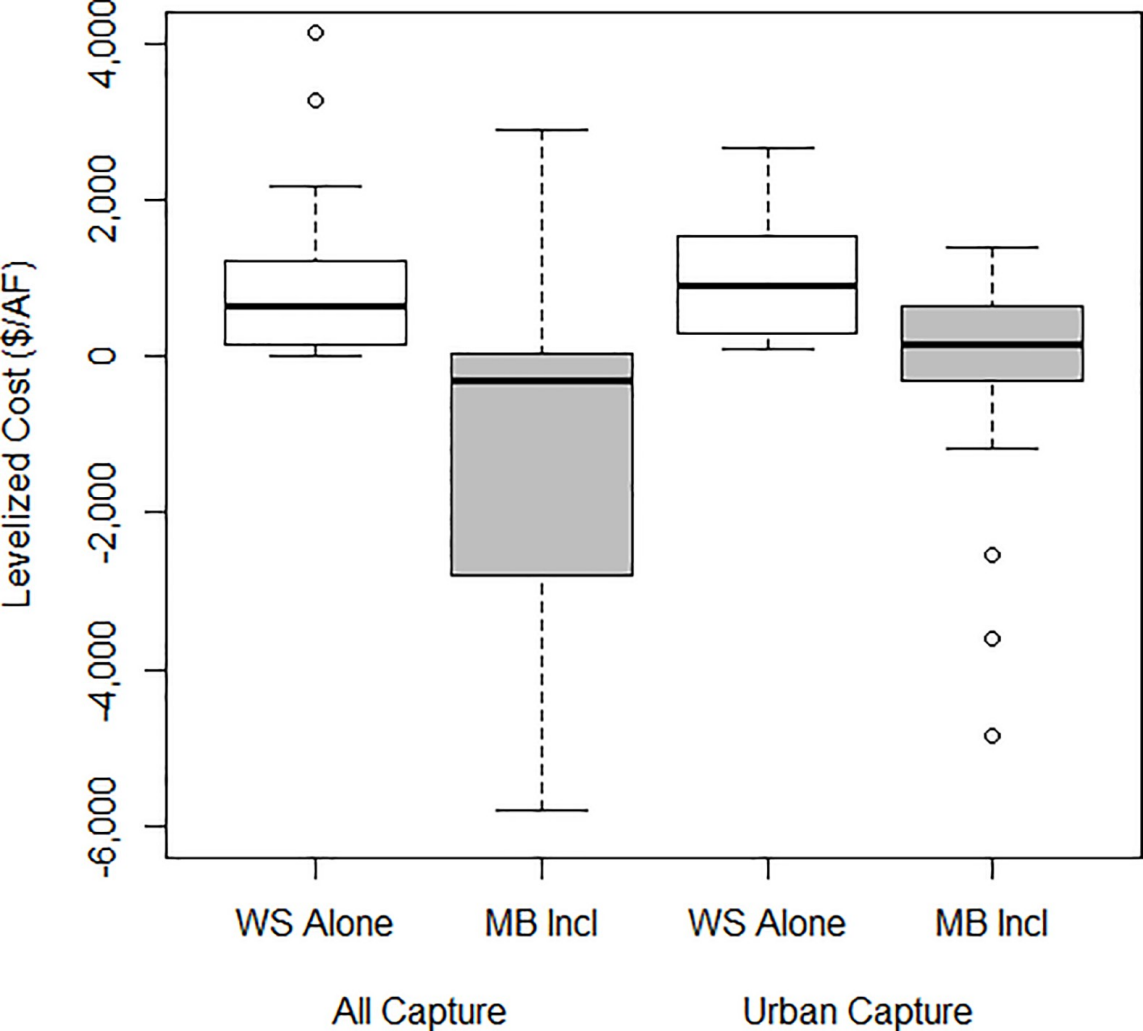
Boxplot of levelized cost of when including water supply (WS) alone in $ per AF (white), compared to net levelized cost incorporating multiple benefits (MB) in $ per AF (grey) for all stormwater capture projects (left) and urban stormwater capture projects (right).

For projects that described additional co-benefits of proposed stormwater projects, the median levelized cost of water was reduced from $807 to–$21.3 per AF for all stormwater capture projects (urban and non-urban) and from $1,030 to $150 for urban stormwater capture projects specifically (Fig 6). The negative levelized cost indicates that the sum of the co-benefits was greater than the total cost of the project.

## Discussion

### Stormwater as a cost-effective water supply

#### Stormwater capture can provide a cost-effective water supply option

The data here demonstrate that stormwater capture presents a cost-effective water supply option, especially when compared with current water supplies. In this dataset, stormwater capture projects had a median levelized cost of $816 per AF (n = 50) and 50% of projects were between $246 and $2,560 per AF. This finding is similar to previous work by Perrone and Rohde (2016) and Cooley and Phurisamban (2019), which demonstrated median costs of stormwater between $590 and $1,550 per AF [8,9]. In comparison, other water supply options often exceed this mean value. For example, previous research has shown that median costs for non-potable reuse are $1,500 per AF, between $1,800 and $2,300 per AF for indirect potable reuse, and $2,800 per AF for seawater desalination [8].

For urban stormwater capture specifically, LID is often considered too expensive to implement. Indeed, this analysis indicates that the levelized cost of decentralized stormwater capture is more expensive on average than centralized stormwater capture. However, the analysis also shows that this is primarily driven by the volume of water captured rather than the specific project mechanisms. For this reason, scaling decentralized stormwater capture projects has the potential to capitalize on similar economies of scale and reduce levelized cost of water. In addition, LID projects, including decentralized stormwater capture, have the potential to scale over time, providing a modular and flexible approach to stormwater management. While the levelized cost of water for these projects ranged widely, the data indicate that projects can be reasonable and competitive options for securing additional water supply.

#### Standardizing analysis for stormwater capture projects

Previous research has quantified the levelized cost of centralized and decentralized stormwater capture; however, methods and data availability have made it challenging to compare projects effectively. Stormwater project costs must consider the life cycle of water from extraction through conveyance, storage, treatment, and delivery. This research demonstrates that stormwater capture projects that include only portions of this process should not be compared directly to determine economic feasibility. This is especially relevant to comparisons between stormwater capture projects that require conveyance and decentralized projects or recharge projects. In this dataset, conveyance projects were significantly more expensive than recharge projects, suggesting that the cost of stormwater capture at scale is largely influenced by the cost of conveying water rather than in developing recharge areas. While decentralized projects were more expensive per acre foot of water supply, by bringing these to scale, there may be opportunities to reduce cost by avoiding conveyance systems.

In addition, quantification of water supply yield should be standardized to the marginal water supply provided by a project. A recent report by the Southern California Water Committee (SCWC) on the cost of stormwater capture in southern California examined 25 retrofit, centralized projects; 4 new, centralized projects; and 3 new, decentralized projects [17]. Because it is challenging to measure the marginal water supply benefit of retrofit projects, the authors estimated the cost of stormwater capture as the marginal cost of the retrofit per the total water supplied by the existing and retrofit systems. This method further incentivizes large, retrofit projects as compared to smaller, distributed projects. Instead, levelized costs must be determined for the marginal water supply increase and compared to the costs of securing alternative water supplies.

Additional research on cost and efficacy will be required to incentivize and scale distributed stormwater infrastructure. Green stormwater infrastructure (or distributed stormwater infrastructure that includes natural elements) has progressed throughout the U.S. as a means of reducing water quality impairments resulting from stormwater runoff. Yet, it is still challenging to measure the resulting change in water supply from these projects. Much of the cost data that are available result from demonstration projects that provide multiple benefits and are not optimized for water supply. As a result, many of these projects are considered too expensive for consideration for alternative water supplies and do not attract the necessary capital for scaling distributed stormwater infrastructure. However, distributed stormwater infrastructure has the potential to provide sustainable water supply at competitive cost, especially if implemented at scale [18]. Rather than focusing on solely water supply, entities should focus on the multiple benefits provided by these projects while advancing research efforts on the efficacy and scalability of distributed infrastructure as a means of providing both water supply and additional co-benefits.

### Maximizing public benefit ofwater supply investments

#### Incorporating multiple benefits

Stormwater capture projects are often part of integrated water management strategies that provide multiple benefits, especially flood control and water quality benefits. Yet current methods for examining the costs of stormwater capture apply the entire cost of the project to a single benefit, dramatically undervaluing integrated projects and incentivizing single-purpose projects that optimize for water supply. In this dataset, projects that considered benefits beyond water supply had a median of 36% reduction in levelized cost of water. In some case, projects demonstrated a negative levelized cost of water when incorporating benefits, indicating that the non-water related benefits are sufficient to justify the project costs alone. Incorporating multiple benefits into the calculation of the cost of water supply can dramatically reduce the levelized cost of stormwater projects, thus incentivizing stormwater capture projects with positive net benefits.

#### Improving guidance to advance analysis of multiple benefits and trade-offs

Funding proposal guidelines play an important role in incentivizing consideration of multiple benefits and trade-offs. For example, guidelines only required proposals to include a limited number of benefits, and thus these analyses likely represent a conservative estimate of multiple benefits for each project. In addition, social impacts should be considered systematically as part of analyses. In each proposal, both quantifiable and non-quantifiable benefits were reported but were not monetized, suggesting additional benefits or trade-offs could provide more information and potentially greater incentive to invest in integrated projects.

Guidelines did not provide substantial direction on quantifying co-benefits or trade-offs of projects, which may have led to over- or under-valuing particular benefits. For example, on average, project proposals for decentralized urban stormwater capture projects on average applied a value of water supply ten times lower than the value of water reported for centralized projects (median value applied of $72 per AF for decentralized compared to $763 per AF for centralized). While there are substantial differences geographically and among agencies on the value of new water supplies, undervaluing the water supplied from distributed stormwater capture can bias towards centralized stormwater capture systems that place greater value on water supply.

These sentiments are echoed in the Strategy to Optimize Resource Management of Stormwater group within the State Water Resources Control Board report, which says that a lack of guidance on how to quantify multiple benefits of projects as a barrier to accelerating stormwater capture in California [19]. Additional tools are becoming available through, for example, triple bottom line analysis that will allow for more in-depth analysis of the net benefit of projects. Additional work is needed within state-wide grant funding to guide analysis and reporting of multiple benefits in order to better examine the net benefits of projects and prioritize funding.

#### Leveraging funding and co-funding opportunities

Projects that achieve multiple benefits provide an opportunity to access funds from additional sources. For example, as demonstrated in Fig 5, many of the urban stormwater projects provided substantial benefits to a variety of agencies, including flood control and energy utilities. While some of the co-benefits identified are more difficult to attribute directly to a local agency or funder (e.g., greenhouse gas emissions reductions), many of the benefits can be attributed to other entities (e.g., flood control or water quality improvements). Incorporating the multiple benefits and costs of stormwater capture into economic evaluations can allow for more transparent comparisons of benefits and costs and help to develop partnerships with entities that benefit directly from the proposed projects.

In the southwestern U.S., climate change will continue to increase scarcity of traditional water supplies [20,21]. Many of the non-urban stormwater capture projects represent implementation or expansion of traditional water management strategies, such as river diversions and dam improvements. In contrast, many of the urban stormwater capture projects, especially decentralized projects, present an opportunity to expand urban water supplies beyond traditional strategies. State-led grant funding through propositions can be used to incentivize innovative stormwater capture projects by prioritizing innovative projects that provide multiple benefits.

#### Avoiding unintended consequences

While stormwater capture is advancing throughout the state, and considered part of more sustainable water management, there are important trade-offs that must be considered when pursuing stormwater capture over other water management options. Porse and Pincetl (2018) demonstrated that increasing stormwater capture and use could significantly reduce stream flows, negatively affecting aquatic habitats and downstream communities [22]. The location and context of the specific projects must be considered as part of analysis on multiple benefits and costs of stormwater capture. In coastal regions, there are substantial opportunities for urban stormwater capture from outfalls that lead directly to the ocean; however, stormwater capture may have a greater number of trade-offs in regions where runoff provides substantial environmental flows. Examining the multiple benefits, as well as the costs and trade-offs of stormwater can provide a better understanding of its viability as an alternative water supply within the context of water supply throughout the state.

## Conclusions

While government agencies, water utilities, and the public are seeking economic information on the cost to capture stormwater, the total cost and the benefits of these projects vary widely and make providing specific cost estimates more challenging. However, this work demonstrates that in California, urban and non-urban stormwater capture may provide economically viable opportunities for increasing local water supply reliability. In addition, scaling distributed stormwater capture has the potential to advance stormwater capture in urban areas while avoiding costly investments in water conveyance systems and allowing for modular, flexible approach to water management.

Beyond California, water managers have the opportunity to expand the number of benefits and trade-offs that are considered in water management decisions. Incorporating multiple benefits into economic evaluations for stormwater capture and additional water management options can dramatically affect the perceived feasibility of these projects. The levelized cost of stormwater capture was dramatically lower when incorporating the reported co-benefits. And, these analyses likely represent the most conservative estimate of multiple benefits for each of these projects. Inclusion of additional benefits will simultaneously provide a better understanding of net benefits of water management and opportunities for developing partnerships around shared outcomes.

Changing precipitation and snowpack patterns throughout the globe are likely to exacerbate water supply challenges in the near future, and California has the potential to serve as a testing ground for advancing alternative water supplies [23,24]. While stormwater management has traditionally focused on improving water quality and reducing flood risk, stormwater capture can provide an alternative water supply in a cost-effective way. Stormwater capture can capitalize on periods of intense rain and advance water supply reliability while improving water quality, reducing flood risk, and providing important environmental and community benefits.

## Supporting information

S1 Dataset (XLSX)
